# Canine hyperactivity, impulsivity, and inattention share similar demographic risk factors and behavioural comorbidities with human ADHD

**DOI:** 10.1038/s41398-021-01626-x

**Published:** 2021-10-01

**Authors:** Sini Sulkama, Jenni Puurunen, Milla Salonen, Salla Mikkola, Emma Hakanen, César Araujo, Hannes Lohi

**Affiliations:** 1 Department of Veterinary Biosciences, University of Helsinki, Helsinki, Finland; 2 Department of Medical and Clinical Genetics, University of Helsinki, Helsinki, Finland; 3 Folkhälsan Research Center, Helsinki, Finland

**Keywords:** ADHD, Psychology

## Abstract

Attention-deficit hyperactivity disorder (ADHD) is a prevalent neurodevelopmental disorder impairing the quality of life of the affected individuals. The domestic dog can spontaneously manifest high hyperactivity/impulsivity and inattention which are components of human ADHD. Therefore, a better understanding of demographic, environmental and behavioural factors influencing canine hyperactivity/impulsivity and inattention could benefit both humans and dogs. We collected comprehensive behavioural survey data from over 11,000 Finnish pet dogs and quantified their level of hyperactivity/impulsivity and inattention. We performed generalised linear model analyses to identify factors associated with these behavioural traits. Our results indicated that high levels of hyperactivity/impulsivity and inattention were more common in dogs that are young, male and spend more time alone at home. Additionally, we showed several breed differences suggesting a substantial genetic basis for these traits. Furthermore, hyperactivity/impulsivity and inattention had strong comorbidities with compulsive behaviour, aggressiveness and fearfulness. Multiple of these associations have also been identified in humans, strengthening the role of the dog as an animal model for ADHD.

## Introduction

Attention-deficit hyperactivity disorder (ADHD) is a highly heritable, childhood-onset neurodevelopmental disorder with an estimated worldwide prevalence of 2–7% in humans [1, 2]. It is characterised by the presence of persistent and inappropriate levels of motor overactivity, impulsivity and inattention [1, 3], caused by abnormalities in attention and reward processing, inhibitory control and emotional regulation [4, 5]. ADHD can be classified into three different presentations: predominantly hyperactive/impulsive, predominantly inattentive or combined type [3]. Often, ADHD persists into adulthood with several comorbidities, such as learning impairments, autism spectrum disorder and anxiety disorders [1, 4], making the disorder a detrimental condition if not diagnosed and treated appropriately.

Impulsivity, a component of human ADHD [3], is characterised by impaired motor inhibitory control and an inability to tolerate gratification delay [6]. It is a dimension of the normal personality continuum observed across species, including humans and dogs [6, 7]. However, high levels of impulsivity are considered abnormal and are also associated with other detrimental behaviours, such as aggression, in several species, including humans [8–10], rodents [11, 12] and dogs [13–17].

Currently, the under-recognition of ADHD hinders its management in humans [1, 18]. This mostly results from the lack of appropriate animal models of ADHD [18]. Studies suggest that 12–15% [19, 20] and 20% [20] of dogs naturally manifest high levels of hyperactivity/impulsivity and inattention, respectively, making the domestic dog a highly potential animal model for ADHD [21–23]. Moreover, these traits are mediated by the same behavioural [21, 24], biological [17, 25] and genetic [24, 26] factors in both dogs and humans, and dogs also respond to medication used to treat ADHD in humans [27, 28]. Furthermore, dogs have even more benefits over the classical animal models, rodents. During domestication, dogs were exposed to similar environmental factors and experienced convergent social evolution [29]. They are also comparable to humans in many complex social cognition tasks [30], genetics [31], body size and physiology and shared environment and lifestyle. The latter feature makes the dog a highly intriguing model for ADHD, as, despite the high heritability estimates, nongenetic factors also contribute to the aetiology of ADHD [32]. However, it is not well known which environmental factors affect ADHD outcomes and to what extent.

To obtain study cohorts truly resembling human ADHD, objective and reliable phenotyping of dogs is required. This can be achieved via owner-filled questionnaires as owners know their dogs’ behaviour well [33] and the reliability of questionnaires is usually good [34, 35]. Based on a survey measuring ADHD in children [36], Vas and colleagues [21] developed an owner-filled dog-ADHD questionnaire, which can reliably measure hyperactivity, impulsivity and attention in dogs. This questionnaire was recently validated, and high levels of impulsivity and inattention were associated with a lower performance in a cognitive task [37].

In this study, we have utilised the same questions developed by Vas et al. [21] as a part of our comprehensive canine behavioural questionnaire. We aimed to examine the demographic, environmental and behavioural factors associated with canine hyperactivity/impulsivity and inattention in a study cohort of over 11,000 Finnish pet dogs. Identification of associated factors could more efficiently help to prevent and manage abnormal levels of hyperactivity/impulsivity and inattention in dogs and could also benefit human ADHD research.

## Material and methods

### Data collection

#### Questionnaire

To collect behavioural and background information from Finnish pet dogs, we designed an online owner-completed behavioural questionnaire. The questionnaire included questions about seven different canine behavioural traits: fear, aggressiveness, noise sensitivity, fear of surfaces and heights, hyperactivity/impulsivity and inattention, separation-related behaviour and compulsive behaviour. Additionally, the questionnaire included a large background section covering demographic and environmental questions related to the dog’s life history. Questionnaire replies were collected from February 2015 to September 2018. The questionnaire and more details about behavioural trait categorisation can be found as Supplementary material in the article by Salonen et al. [20]. This current study is based on the same data as the article by Salonen et al. [20] but here we utilised large multivariate analyses. Here, we studied the demographic and environmental factors associated with hyperactivity/impulsivity and inattention.

#### Hyperactivity/impulsivity and inattention

To measure individual differences in hyperactivity/impulsivity and inattention, we used the dog ADHD survey developed and validated by Vas et al. [21], which is based on a validated parent-report rating scale measure of ADHD and related problems in children (the ADHD RS Parent version [36]). The survey includes 13 statements (items) (Supplementary Table S1) concerning hyperactive, impulsive and inattentive behaviour, which were translated into Finnish. The dog owners were asked to answer how often the statement is true for their dog on a four-point Likert scale (1 = never, 2 = rarely, 3 = often, 4 = very often). Paralleling the original factor structure of Vas et al. [21], a principal component analysis with a promax rotation divided the questionnaire statements into two components, hyperactivity/impulsivity and inattention, which consisted of five and seven statements, respectively. One statement (Item 11: 'It is likely to react hastily and that’s why it is failing tasks') loaded equally on both components and was thus excluded from the analysis. We calculated component scores of hyperactivity/impulsivity and inattention for each dog, with higher component scores indicating a higher level of hyperactivity/impulsivity or inattention. Detailed information about the statements and components can be found in Supplementary Material in the article of Salonen et al. [20]. The Finnish translation of the questionnaire was recently validated in Salonen et al. [38].

### Demographic, behavioural and environmental variables

Before statistical analyses, we edited some demographic and environmental variables derived from the behavioural questionnaire. We created some new variables already described in Puurunen et al. [39], Hakanen et al. [40] and Mikkola et al. [41]. All explanatory variables derived from the behavioural questionnaire are explained in detail in Supplementary Table S2.

Briefly, we included 22 breeds with adequate sample size and mixed breed dogs and grouped other breeds under breed group other. A new variable 'body size' was created by assessing the average heights of breeds and categorised the dogs into small, medium and large dogs (Supplementary Methods). As body size could not be determined for mixed breed dogs, they were excluded when body size was included in the analysis. To quantify the environmental land-use in the dog’s home place, we generated a continuous variable 'urban environment score' (Supplementary Methods), with a higher urban environment score indicating a higher proportion of built environment.

Additionally, we created three categorical behavioural variables: 'compulsive behaviour', 'aggressiveness' and 'fearfulness' (Supplementary Methods). In all three traits, dogs were divided into three groups: low, moderate and high. The low group included dogs that never showed compulsive, aggressive or fearful behaviour, the moderate group included dogs that showed these behaviours no more than occasionally, and the high group included the dogs with regular compulsive, aggressive or fearful behaviour at least in one subtrait.

### Statistical analyses

All statistical analyses were conducted in R version 3.6.2 [42]. Generalised linear models were used to analyse the associations between demographic, environmental and behavioural variables and hyperactivity/impulsivity and inattention. Gamma distribution with a log link function was used in both hyperactivity/impulsivity and inattention models, as it best fitted the data. The initial data consisted of 13,715 dogs in 264 breeds. After excluding individual dogs with missing or incomplete responses in the studied explanatory variables, the data included 6,400 dogs in both hyperactivity/impulsivity and inattention.

We used the hyperactivity/impulsivity and inattention component scores as continuous response variables in the analyses. Fourteen explanatory variables were selected for the analyses based on previous literature. Age, sex, sterilisation, breed and body size were included as demographic explanatory variables, weaning age, activities/training, daily exercise, owner’s dog experience, daily time spent alone and urban environment score as environmental explanatory variables, and compulsive behaviour, fearfulness and aggressiveness as behavioural explanatory variables.

We used a forward stepwise AIC (Akaike Information Criterion) model selection to find the best fitting model starting with a model including age and sex as explanatory variables. Sexes and age groups differed in prevalence in our previous study [20]. The AIC model selection and the final models are presented in Supplementary Table S3. To maximise sample sizes, we created new subsets of the initial data after model selection by including all dogs that had missing responses in the explanatory variables that were not selected in the final models. As a result, the final datasets consisted of 11,539 and 11,164 individuals in hyperactivity/impulsivity and inattention, respectively.

Model fit was assessed carefully (Supplementary Methods). After fitting the model, estimated marginal means were calculated with the package 'emmeans' [43] to obtain the adjusted means and confidence limits for categorical explanatory variables. The package 'effects' [44] in R was used to obtain the effects of continuous explanatory variables, adjusting for other variables in the models. Analysis of variance (ANOVA) was conducted with the package 'car' [45] in R to get the overall effects of the explanatory variables on hyperactivity/impulsivity and inattention scores.

Based on previous studies, we formed several a priori contrasts between the levels of explanatory variables. We hypothesised that younger dogs are more hyperactive and impulsive and more inattentive than older dogs [21, 22, 46–48]. Besides, in both hyperactivity/impulsivity and inattention, we hypothesised that dogs participating in activities or training often (at least weekly) would differ from dogs participating in activities or training never or seldom [21, 22, 49]. In hyperactivity/impulsivity, we hypothesised that large dogs would differ from small dogs in their hyperactivity/impulsivity [21, 48]. Furthermore, we hypothesised that dogs in high compulsive behaviour, high aggressiveness and high fearfulness groups would be more hyperactive and impulsive than dogs in low compulsive behaviour, low aggressiveness and low fearfulness groups, respectively [19].

The package 'emmeans' [43] in R was used to examine all pairwise comparisons between the levels of the categorical explanatory variables and to examine the a priori contrasts. We corrected *p* values for false discovery rate (FDR), except contrasts chosen a priori. We set the significance cut-off at *p* value <0.05.

## Results

### Study cohort and demographics

We studied the effects of environmental, demographic and behavioural factors on hyperactivity/impulsivity and inattention in study cohorts of 11,539 and 11,164 dogs, respectively. The hyperactivity/impulsivity score varied from −1.62 to 5.23 (mean −0.01) and the inattention score from −1.81 to 4.81 (mean −0.01). In both hyperactivity/impulsivity and inattention, 51% of the dogs were females. The age of the dogs varied from 2 months to 17.9 years, with a mean of 4.7 years in both traits. More detailed demographics are presented in Supplementary Table S4.

### Factors associated with hyperactivity/impulsivity

Several demographic, environmental and behavioural variables, including age, sex, breed, body size, daily exercise, daily time spent alone, owner’s dog experience, compulsive behaviour, aggressiveness and fearfulness were associated with canine hyperactivity/impulsivity (Table 1).

**Table 1:** Associations of the demographic, environmental and behavioural variables with hyperactivity/impulsivity and inattention scores in the generalised linear models with gamma distribution and log link function.

Variable	Hyperactivity/impulsivity			Inattention		
	*F*	*p* value	DF	*F*	*p* value	DF
Age	295.93	<0.0001*	1	8.73	0.0031*	1
Age^2				0.56	0.5607	1
Sex	68.39	**<0.0001**	1	80.03	**<0.0001**	1
Breed	4.37	**<0.0001**	22	11.23	**<0.0001**	23
Body size	11.32	**0.0004**	2			
Daily exercise	9.69	**0.0001**	4			
Daily time spent alone	8.62	**0.0004**	3	5.27	**0.0046**	3
Owner’s dog experience	21.53	**0.0001**	1			
Activities/training				63.77	**<0.0001**	2
Fearfulness	202.57	**<0.0001**	2	112.79	**<0.0001**	2
Aggressiveness	60.93	**<0.0001**	2	36.03	**<0.0001**	2
Compulsive behaviour	241.21	**<0.0001**	2	160.01	**<0.0001**	2

*P* values are controlled for false discovery rate if no a priori contrasts were set before analyses. Variables for which a priori contrasts were set and which *p* values are not false discovery controlled are denoted with *. Significant effects are indicated in bold (*p* value <0.05). *N* = 11,539 (hyperactivity/impulsivity) and *N* = 11,164 (inattention).

**Fig. 1:**
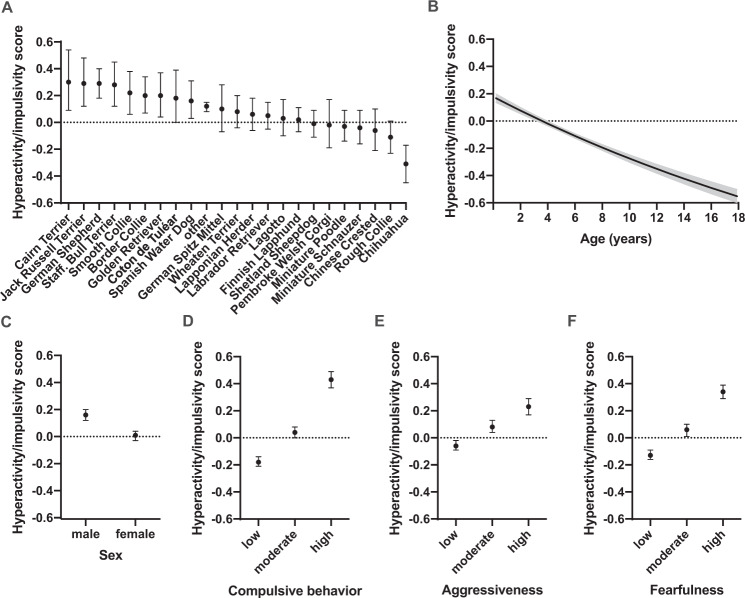
Canine hyperactivity/impulsivity. The effects of breed (**A**), age (**B**), sex (**C**), compulsive behaviour (**D**), aggressiveness (**E**) and fearfulness (**F**) on canine hyperactivity/impulsivity. Grey area (**B**) and error bars (**A**, **C**, **D**, **E**, **F**) indicate 95% confidence limits. *N* = 11,539.

We examined breed differences in hyperactivity/impulsivity in 23 breeds, including other groups consisting of all the other breeds in the data. We detected significant differences in hyperactivity/impulsivity scores between dog breeds. The breeds with the highest scores were Cairn Terrier, Jack Russell Terrier, German Shepherd and Staffordshire Bull Terrier. The breeds with the lowest scores were Chinese Crested Dog, Rough Collie and Chihuahua (Fig. 1A). The largest pairwise differences were found between Chihuahua and German Shepherd Dog (z-ratio = −6.07, df = 1, *p* = 0.0008), Chihuahua and Jack Russell Terrier (z-ratio = −5.41, df = 1, *p* = 0.0008), Chihuahua and Staff. Bull Terrier (z-ratio = −5.35, df = 1, *p* = 0.0008) and Chihuahua and other (z-ratio = −5.22, df = 1, *p* = 0.0008). All pairwise breed differences are presented in the Supplementary Dataset.

Both the age and the sex of the dog were associated with hyperactivity/impulsivity (Table 1, Supplementary Table S5, and Fig. 1B, C). Hyperactivity/impulsivity scores were highest in young dogs (*F* = 295.93, df = 1, *p* < 0.0001) as hypothesised a priori. Male dogs had higher hyperactivity/impulsivity scores than females (z-ratio = 8.27, df = 1, *p* < 0.0001). There was also an association between body size and hyperactivity/impulsivity (Supplementary Table S5 and Supplementary Fig. S1A). Medium-sized dogs had higher hyperactivity/impulsivity scores than small (z-ratio = 4.74, df = 1, *p* = 0.0008) or large (z-ratio = 2.78, df = 1, *p* = 0.0224) dogs, and, as we hypothesised in our a priori contrast, there was also a significant difference between small and large dogs (z-ratio = −2.40, df = 1, *p* = 0.0166).

Dogs getting less daily exercise and spending more time alone had higher hyperactivity/impulsivity scores (Supplementary Table S5 and Supplementary Fig. S1B, C). Dogs getting less than 1 h of exercise per day had higher hyperactivity/impulsivity scores than dogs exercising more than 3 h (z-ratio = 4.75, df = 1, *p* = 0.0008), 2–3 h (z-ratio = 3.96, df = 1, *p* = 0.0008), or 1–2 h (z-ratio = 2.5, df = 1, *p* = 0.0416) per day. Additionally, dogs exercising 1–2 h had higher hyperactivity/impulsivity scores when compared with dogs exercising 2–3 h (z-ratio = 2.66, df = 1, *p* = 0.0297) or more than 3 h (z-ratio = 3.74, df = 1, *p* = 0.0014) daily. Dogs that spent alone more than 8 h daily had higher hyperactivity/impulsivity scores than dogs spending less than 3 h (z-ratio = 3.99, df = 1, *p* = 0.0008), 3–6 h (z-ratio = 4.83, df = 1, *p* = 0.0008) or 6–8 h (z-ratio = 3.48, df = 1, *p* = 0.0031) alone. The owner’s dog experience was associated with higher hyperactivity/impulsivity scores (Supplementary Table S5 and Supplementary Fig. S1D). If the dog was not the owner’s first dog, it was more likely to have a higher hyperactivity/impulsivity score than if the dog was the owner’s first dog (z-ratio = 4.65, df = 1, *p* = 0.0001).

Compulsive, aggressive and fearful dogs were reported to have higher hyperactivity/impulsivity scores (Supplementary Table S5 and Fig. 1D, E, F). As we hypothesised a priori, dogs showing high levels of compulsive behaviour had higher hyperactivity/impulsivity scores when compared with dogs showing low levels of compulsive behaviour (z-ratio = 21.50, df = 1, *p* < 0.0001). Also, dogs showing high levels of compulsive behaviour had higher hyperactivity/impulsivity scores than dogs showing moderate levels of compulsive behaviour (z-ratio = 13.78, df = 1, *p* = 0.0008), and dogs showing moderate levels of compulsive behaviour had higher hyperactivity/impulsivity scores than dogs showing low levels of compulsive behaviour (z-ratio = 11.61, df = 1, *p* = 0.0008). Similarly, as we hypothesised a priori, dogs showing high levels of aggressiveness had higher hyperactivity/impulsivity scores when compared with dogs showing low levels of aggressiveness (z-ratio = 10.21, df = 1, *p* < 0.0001). Dogs showing high levels of aggressiveness had also higher hyperactivity/impulsivity scores than dogs showing moderate levels of aggressiveness (z-ratio = 4.71, df = 1, *p* = 0.0008), and dogs showing moderate levels of aggressiveness had higher hyperactivity/impulsivity scores than dogs showing low levels of aggressiveness (z-ratio = 6.80, df = 1, *p* = 0.0008). Furthermore, as also hypothesised a priori, dogs showing high levels of fearfulness had higher hyperactivity/impulsivity scores when compared with dogs showing low levels of fearfulness (z-ratio = 20.01, df = 1, *p* < 0.0001). Dogs showing high levels of fearfulness had also higher hyperactivity/impulsivity scores than dogs showing moderate levels of fearfulness (z-ratio = 10.93, df = 1, *p* = 0.0008), and dogs showing moderate levels of fearfulness had higher hyperactivity/impulsivity scores than dogs showing low levels of fearfulness (z-ratio = 8.77, df = 1, *p* = 0.0008).

### Factors associated with inattention

Statistical analysis identified several demographic, environmental and behavioural factors associated with inattention scores, including breed, age, sex, daily time spent alone, activities/training, compulsive behaviour, aggressiveness and fearfulness (Table 1).

**Fig. 2:**
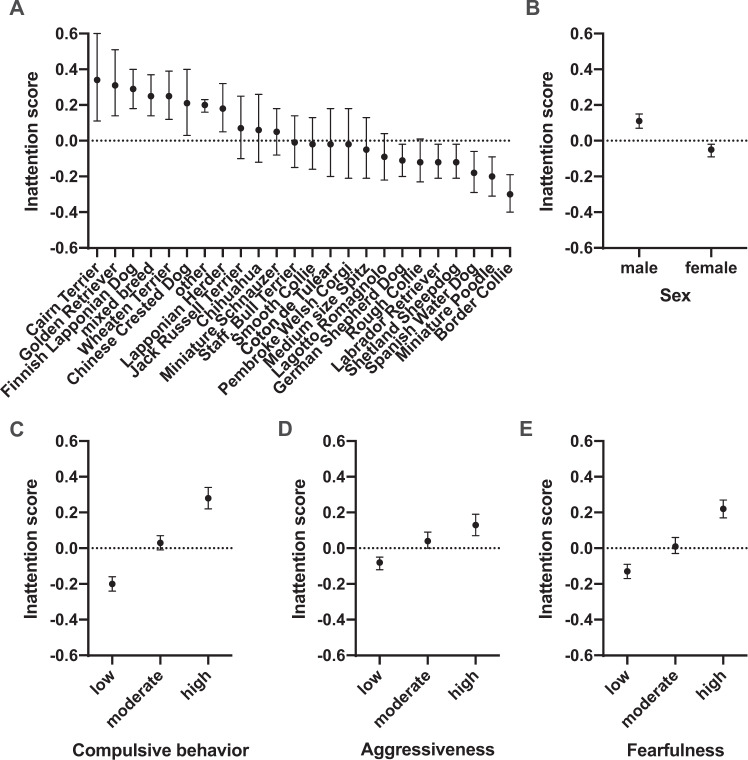
Canine inattention. The effects of breed (**A**), sex (**B**), compulsive behaviour (**C**), aggressiveness (**D**) and fearfulness (**E**) on canine inattention. Error bars indicate 95% confidence limits. *N* = 11,164.

We detected significant differences in inattention scores between dog breeds. The breeds with the highest scores were Cairn Terrier, Golden Retriever and Finnish Lapponian Dog. The breeds with lowest scores were Spanish Water Dog, Miniature Poodle and Border Collie (Fig. 2A). The largest pairwise differences were found between Border Collie and other (z-ratio = −8.01, df = 1, *p* = 0.0005), Border Collie and Finnish Lapponian Dog (z-ratio = −7.60, df = 1, *p* = 0.0005), Border Collie and mixed breed (z-ratio = −6.83, df = 1, *p* = 0.0005) and Border Collie and Wheaten Terrier (z-ratio = −6.54, df = 1, *p* = 0.0005). All pairwise breed differences are presented in the Supplementary Dataset.

Age of the dog was associated with inattention (Table 1 and Supplementary Fig. S2A). Inattention scores were highest in young dogs (linear effect: *F* = 8.73, df = 1, *p* = 0.0031, quadratic effect: *F* = 0.56, df = 1, *p* = 0.561) as we hypothesised a priori. Significant differences were also found between sexes as males had higher inattention scores than females (z-ratio = 8.95, df = 1, *p* < 0.0001; Supplementary Table S6 and Fig. 2B).

Dogs spending more time alone and participating less frequently in activities and training had higher inattention scores (Supplementary Table S6 and Supplementary Fig. S2B, C). Dogs that spent alone more than 8 h daily had higher inattention scores than dogs that spent alone less than 3 h (z-ratio = 2.99, df = 1, *p* = 0.0097), 3–6 h (z-ratio = 3.85, df = 1, *p* = 0.0005) or 6–8 h (z-ratio = 2.49, df = 1, *p* = 0.0327) per day. As we hypothesised a priori, there was a difference between dogs that participate in activities and training never/seldom or at least weekly, since dogs participating in activities and training never/seldom had higher inattention scores (z-ratio = 9.52, df = 1, *p* < 0.0001). Dogs participating in activities sometimes had higher inattention scores than dogs that trained weekly (z-ratio = 9.28, df = 1, *p* = 0.0005).

The dogs with compulsive, aggressive or fearful behaviour also had higher inattention scores (Supplementary Table S6 and Fig. 2C, D, E). Dogs showing high levels of compulsive behaviour had higher inattention scores when compared with dogs showing low (z-ratio = 17.04, df = 1, *p* = 0.0005) or moderate (z-ratio = 8.63, df = 1, *p* = 0.0005) levels of compulsive behaviour. Dogs showing moderate levels of compulsive behaviour also had higher inattention scores than dogs showing low levels of compulsive behaviour (z-ratio = 12.17, df = 1, *p* = 0.0005). Similarly, dogs showing high levels of aggressiveness had higher inattention scores when compared with dogs showing low (z-ratio = 7.50, df = 1, *p* = 0.0005) or moderate (z-ratio = 2.72, df = 1, *p* = 0.0199) levels of aggressiveness. Dogs showing moderate levels of aggressiveness had higher inattention scores than dogs showing low levels of aggressiveness (z-ratio = 5.93, df = 1, *p* = 0.0005). Additionally, dogs showing high levels of fearfulness had higher inattention scores when compared with dogs showing low (z-ratio = 14.86, df = 1, *p* = 0.0005) or moderate (z-ratio = 7.90, df = 1, *p* = 0.0005) levels of fearfulness. Dogs showing moderate levels of fearfulness had higher hyperactivity/impulsivity scores than dogs showing low levels of fearfulness (z-ratio = 6.85, df = 1, *p* = 0.0005).

## Discussion

We have performed the most extensive survey-based study on canine hyperactivity/impulsivity and inattention with over 11,000 dogs, identifying many associated demographic, environmental and behavioural factors. This study is based on the same data as our previous exploration [20] but we used a more comprehensive and precisive approach here, including complex multivariate models and hyperactivity/impulsivity and inattention scores as continuous variables. We confirmed the behavioural associations observed in our previous study [20]. In addition, we report novel demographic and environmental associations, observed notable breed differences in the presentation of the phenotypes, and note a significant overlap with the reported risk factors and comorbidities in human ADHD.

Multiple demographic factors were associated with hyperactivity/impulsivity and inattention in dogs, including age, sex and body size. Hyperactivity/impulsivity and inattention were most common in young dogs. Both traits are attenuated with age, but hyperactivity/impulsivity slightly more than inattention. Earlier research has also demonstrated that hyperactive and impulsive [22, 46–48] and inattentive [21, 22, 47] behaviour are much more prevalent in young dogs. However, Vas et al. [21] did not find a significant association between impulsivity and the dog’s age. In our study, hyperactivity/impulsivity and inattention were more prevalent in male than female dogs. This result contradicts earlier studies that have found no significant difference between the sexes [48, 50] or have identified females as more hyperactive/impulsive [22]. Only Vas et al. [21] found males to be more impulsive than females, but the difference was not statistically significant. Human ADHD is similarly a childhood-onset disease and more common in boys than girls [1, 51]. However, the reason for this sex difference is unclear. Girls are more often affected by the predominantly inattentive subtype of ADHD [52] and show less visible signs of ADHD [53]. However, girls may require a higher burden of genetic risk factors to manifest ADHD [54]. A role of steroid hormones has also been proposed, as exposure to high levels of testosterone during gestation might affect the dopaminergic system, and thus potentially predispose boys to ADHD [55, 56]. These prenatal hormonal effects have not yet been studied in dogs.

Previous research suggests that small dogs are more impulsive [48]. In contrast, we observed higher hyperactivity/impulsivity scores in medium-sized and large dogs than in small dogs. Wright, Mills & Pollux [48] suggested their findings to result from multiple active small terrier breeds. Similarly, our study included several medium-sized working dog breeds, potentially explaining the observed association between higher impulsivity and medium body size. These breeds have been bred for increased activity, alertness and vigilance to maximise their properties as working dogs [28]. When adjusting the model for body size, the most hyperactive/impulsive breeds in our study included dogs from all sizes and many breeds differed significantly from each other. Thus, the differences between breeds are not explained only by their size differences, and this result also indicates a genetic origin for hyperactivity/impulsivity.

We analyzed hyperactivity/impulsivity and inattention in more than 20 different breeds and found considerable differences. Cairn Terrier, Jack Russell Terrier, German Shepherd Dog, Staffordshire Bull Terrier and Smooth Collie had the highest hyperactivity/impulsivity scores whereas Chihuahua, Rough Collie, Chinese Crested Dog, Miniature Schnauzer and Poodle had the lowest scores. In inattention, Cairn Terrier, Golden Retriever, Finnish Lapponian Dog, mixed breed and Wheaten Terrier had the highest scores, and Border Collie, Poodle, Spanish Water Dog, Shetland Sheepdog and Labrador Retriever lowest scores. Different behaviour traits are valued in breeds used for various purposes, and thus, selective breeding in dogs has influenced their breed-typical behaviour [57, 58]. For example, in some working dog breeds, such as German Shepherd Dog and Border Collie, high activity, impulsivity and attention are favoured. These dogs usually have better trainability and working ability due to higher attention spans and reactivity [59]. On the contrary, these traits are not favoured in breeds which are now preferred as a pet or show dogs, such as Chihuahua, Rough Collie and Poodle, since less active and impulsive dogs are more easy companions in a less active way of life. But, as a side-effect, inattentive behaviour can be enriched in these breeds.

Interestingly, in our study, Smooth Collie was one of the breeds with the highest hyperactivity/impulsivity scores, whereas Rough Collie was one with the lowest scores. Except for coat length, these two breeds are almost identical. However, the use of these breeds nowadays differs. After Lassie movies, the popularity of Rough Collies as companion dogs increased whereas Smooth Collies have never achieved a high level of popularity and are still often used as working dogs and in dog sports [60]. This might also explain the observed difference in hyperactivity/impulsivity between these closely related breeds. Unfortunately, a comprehensive comparison of our results to earlier findings is difficult as breed groups instead of individual breeds are used in many previous studies [22, 47, 50].

We identified several environmental factors associated with hyperactivity/impulsivity and inattention. Low daily exercise and rare participation in activities and training were associated with higher hyperactivity/impulsivity scores and higher inattention scores, respectively. Ley et al. [47] found that dogs spending less time inside were more active than dogs spending more time indoors. However, they identified a significant positive correlation between dogs’ age and the time spent inside, suggesting that the observed association might be affected by the fact that older dogs usually spent more time indoors than young dogs. Several studies have shown that dogs that are trained often are less inattentive than dogs trained less frequently [21, 22, 49]. Exercise and enrichment can be ways to fulfil the species-specific needs of dogs. Thus, dogs exercising more and participating more frequently in activities and training can release their energy and frustration in a controlled manner. Therefore, in some cases, high levels of hyperactivity/impulsivity and inattention may be due to limited possibilities to release energy and reduce activity levels. However, it is also possible that owners are not willing to train and participate in activities with inattentive dogs, as training with them can be uncomfortable and unsatisfactory due to the dog’s concentration difficulties. Studies investigating the effects of exercise on human ADHD are sparse and have small sample sizes, but in children with ADHD, a few meta-analyses demonstrate the physical exercise to alleviate hyperactivity, impulsivity and inattention to some extent [61, 62].

We observed a novel finding indicating that dogs spending more time alone daily had higher hyperactivity/impulsivity and inattention scores than dogs that spent less time alone. As dogs are social animals, they can be stressed or frustrated when left alone for a long time. This stress and frustration may erupt as hyperactive, impulsive and inattentive behaviour. Dogs generally remain calm and rest during the period of separation from their owners. More extended separation may result in more energetic behaviour and greater physical activity when the owner returns, potentially reflecting dogs’ increased social isolation during the prolonged separation period. Rehn & Keeling [63] found that after longer separations dogs tended to offer more intense greeting behaviours, with a higher frequency of physical activity and attention behaviour, confirming the effect of time left alone. However, it is also possible that dogs spending more time alone also otherwise get less attention and exercise from their owners. No further conclusions about the relationship between the time spent alone and more hyperactive/impulsive and inattentive behaviour can be drawn, and the causality can only be speculated.

As a novel finding, we discovered that hyperactivity/impulsivity is more common in dogs that are not their owners’ first dogs. As no previous results report a similar association, we can only speculate about the possible relationship between these factors. People may try to choose easy individuals from less active breeds, like companion dog breeds as their first dogs. When they gain experience handling a dog, they may feel more comfortable choosing individuals from more active and challenging breeds, such as herding breeds, as their following dogs. Furthermore, with their first dogs, people may try dog sports and hobbies and if they get excited about the particular sport, they may choose their following dogs from more active and athletic breeds to be more successful in that sport. It is also possible that owners with more than just one dog are more experienced and can better recognise different behaviours, such as hyperactivity/impulsivity and inattention, in their dogs.

Interestingly, both hyperactivity/impulsivity and inattention scores were significantly higher in dogs with high levels of compulsive behaviour, aggressiveness and fearfulness. Impulsivity has been considered in relation to aggressiveness for a long time in dogs [13–15, 19]. One recent study discovered a connection between compulsive behaviour and hyperactivity, and between fearfulness and hyperactivity [19]. Furthermore, Wright et al. [48] described that impulsivity was more common in dogs reported to have other behavioural problems. Still, they did not declare what these behavioural problems were. However, inattention has been little studied in dogs and these observed comorbidities (between inattention and compulsive behaviour, aggressiveness and fearfulness) have been previously described in dogs only in our previous exploration [20] of this same but expanded dataset. In this present study, these comorbidities persisted after the inclusion of several demographic and environmental variables in the same multivariate model.

Paralleling our results, ADHD is known to have several behavioural comorbidities, such as autism spectrum disorder, learning impairments, and anxiety and mood disorders in humans [1, 4, 64]. Impulsivity, a key component of ADHD, is also associated with aggressive behaviour [8]. This can often be classified as impulsive or reactive aggression [65–67]. Furthermore, obsessive-compulsive disorder (OCD) often co-occurs with ADHD in humans, with both conditions characterised by impaired inhibitory control and deficit in executive function [64, 68–72].

The comorbid association between hyperactivity/impulsivity and aggressiveness, fearfulness and compulsive behaviour may refer to shared underlying neurobiological pathways and brain structures involved in these traits. ADHD and impulsive behaviour are associated with deficits in the frontostriatal circuit and abnormal levels of activation in, for example, prefrontal cortex (PCF), anterior cingulate cortex (ACC) and striatum [64, 70, 73]. Similarly, OCD is also characterised by abnormal frontostriatal circuit activity and likewise, involves the striatum, PFC and ACC [71, 72, 74, 75] Besides ADHD and OCD, aggressiveness also involves the brain reward system and neurological pathways involved in aggressiveness similarly connect to PFC and striatum [65, 67]. Finally, fear and anxiety are also associated with activity in PFC and ACC [76, 77].

Our results indicate that different breeds may be useful in modelling the different presentations of ADHD. Cairn Terrier could be a suitable model for ADHD as it has a high mean score in both hyperactivity/impulsivity and inattention traits, together with compulsive behaviour and aggression comorbidities. In contrast, Labrador Retriever had a low mean score in both traits and they also display comorbid behaviours fearfulness, aggression and compulsive behaviour very rarely. Spanish Water Dog had a low mean score in inattention, but a high mean score in hyperactivity/impulsivity, together with comorbidities, such as fearfulness. In contrast, Chinese Crested Dog had a high mean score in inattention, but a low mean score in hyperactivity/impulsivity as well as high levels of fearfulness, aggression and compulsive behaviour. Finally, within-breed studies would be helpful in revealing the genetic and biological factors associated with hyperactivity/impulsivity and inattention.

This study has limitations. Our analysis is based on a questionnaire and owners’ participation in the study was voluntary. Questionnaires can be an effective way to collect data as their reliability has been useful in behavioural science and questionnaire answers are strongly linked to the behaviour of the animals [34, 37]. However, questionnaires can be subjective. Our data is a self-selected convenience sample and may not represent the overall dog population in Finland. Due to missing data, several dogs were excluded from the analyses and thus, future studies should aim to collect more complete data. Finally, some of the breeds we studied included both working and show lines and we could not separate the lines within the breeds. In our future studies, we aim to collect information about the line of the dog to assess the possible behaviour differences between the show and working lines.

In conclusion, we show that canine hyperactivity/impulsivity and inattention are associated with several demographic, environmental and behavioural factors. Our results also suggest that these traits have a strong genetic basis. Furthermore, our results reinforce the dog as an appropriate, up-and-coming animal model of ADHD. Hyperactivity/impulsivity and inattention were more common in young and male dogs, and the same age and sex effects are well established in human ADHD as well. Additionally, similar behavioural comorbidities in canine hyperactivity/impulsivity and inattention and human ADHD strengthen the hypothesis of the shared neurobiological pathways behind these traits in both species. Furthermore, the similarities in genetics, physiology and living environment between dogs and humans make the dog a more intrinsic model to ADHD than, for example, rodents. Therefore, understanding the factors that affect canine hyperactivity/impulsivity and inattention can benefit not only recognition and management of these traits in dogs but also human ADHD research.

## Supplementary information

Supplementary methods

Supplementary tables

## Data Availability

The anonymized data is available as Supplementary material in the article by Salonen et al. [20].

## Code availability

The R environment is freely available at https://www.r-project.org/. Additionally, all the R packages utilised for the analyses are freely available at https://cran.r-project.org/web/packages/available_packages_by_name.html. Specific R scripts used for the analyses are available from the corresponding author on reasonable request.
