# Supplementary material for: Canine hyperactivity, impulsivity, and inattention share similar demographic risk factors and behavioural comorbidities with human ADHD
Source: Transl Psychiatry. 2021 Oct 1;11:501. doi: 10.1038/s41398-021-01626-x (PMC8486809; doi:10.1038/s41398-021-01626-x)
Supplement: Supplementary file 1 — Supplementary methods [file 41398_2021_1626_MOESM1_ESM.doc]

**Canine** **hyperactivity, impulsivity, and inattention share similar demographic risk factors and behavioural comorbidities with human ADHD**

Sini Sulkama, MSc*^1,2,3^, Jenni Puurunen, PhD*^1,2,3^, Milla Salonen, PhD^1,2,3^, Salla Mikkola, MSc^1,2,3^, Emma Hakanen, MSc^1,2,3^, César Araujo, PhD^1,2,3^, Hannes Lohi, PhD, prof.^1,2,3#^

^1^Department of Veterinary Biosciences, University of Helsinki, Helsinki, Finland

^2^Department of Medical and Clinical Genetics, University of Helsinki, Helsinki, Finland

^3^Folkhälsan Research Center, Helsinki, Finland

* equal contribution

#  Corresponding author:

Hannes Lohi, PhD, Professor

+358294125085

Email: hannes.lohi@helsinki.fi (HL)

PL 63 (Haartmaninkatu 8), 00014 HELSINGIN YLIOPISTO, Finland

**Supplementary Methods**

**Demographic, behavioural and environmental variables**

A new categorical variable “body size” was created by assessing the average heights of breeds based on FCI and AKC breed standards, when available. We categorised the dogs in three body size categories: small (height ≤ 35 cm), medium (average height 36-49 cm), and large (average height ≥ 50 cm) based on the breed’s average height.

We quantified the environmental land-use in the dog’s home place and based on this land-use, we generated a continuous variable “urban environment score”. The proportion of three different land-use types (artificial surfaces, agricultural areas, and forests and semi-natural areas) within a three-kilometre range was defined using a public land-use database CORINE2012 with a 25-m resolution. The land-use information was simplified and reduced to a continuous rural-urban gradient using principal component analysis. A higher proportion of built environment correlated with a higher urban environment score.

We created three categorical behavioural variables: “compulsive behaviour”, “aggressiveness”, and “fearfulness”. All of these traits included subtraits and the behavioural variables “compulsive behaviour”, “aggressiveness”, and “fearfulness” were combinations of all subtraits of each trait. In “compulsive behaviour” dog owners were asked to estimate the occurrence of tail chasing, reflection/shadow snatching, surface licking, pacing, staring, the time dog spends near water bowl (indicating water bowl compulsion), and self-biting. “Fearfulness” consisted of three fear subtraits: fear towards strangers, other dogs, and novel situations. Dog owners were asked how often their dog showed fear in these situations. In “aggressiveness” owners were asked to rate the likelihood of their dog displaying aggressive behaviour towards strangers and family members. The signs of aggressive behaviour were snapping or biting and growling. In all three traits, dogs were divided into three groups: low, moderate, and high. The low group included dogs that never showed compulsive, aggressive, or fearful behaviour, the moderate group included dogs that showed these behaviours no more than occasionally, and the high group included the dogs with regular compulsive, aggressive, or fearful behaviour at least in one subtrait.

**Statistical analyses**

Model fit was assessed carefully. Firstly, we fitted generalised additive models with the package “gam” ^1^ in R to check the linearity assumptions of continuous explanatory variables. We included both linear and quadratic variables (e.g. age and age^2^) in the model if the assumption was not met. Secondly, we plotted standardised residuals using the packages “ggplot2” ^2^ and “boot” ^3^ in R, tested the distribution of residuals with the package “rcompanion” ^4^ in R, and inspected the possible outliers with the packages “broom” ^5^ and “dplyr” ^6^ in R. We found some outliers in the models, but their removal did not affect the estimates, and as they were real data points, they were kept in the final models. We evaluated the presence of heteroscedasticity from the residual plots. Based on these plots, variances between groups were similar. Finally, we tested multicollinearity by requesting the generalised variance inflation factor (gVIF) with the package “car” ^7^ in R.

**Supplementary references**

1. Hastie, T. gam: Generalized Additive Models. (2018). Available at: https://cran.r-project.org/packages/=broom.

2. Wickham, H. ggplot2: Elegant Graphics for Data Analysis. *Springer-Verlag New York* (2016). Available at: https://ggplot2.tidyverse.org. (Accessed: 29th May 2019)

3. Canty, A. & Ripley, B. *boot: Bootstrap R (S-Plus) Functions*. *R package version 1.3*–*4* **1** (2012).

4. Mangiafico, S. rcompanion: Functions to Support Extension Education Program Evaluation. (2019). Available at: https://cran.r-project.org/package=rcompanion. (Accessed: 29th May 2019)

5. Robinson, D. & Hayes, A. broom: Convert Statistical Analysis Objects into Tidy Tibbles. (2018). Available at: https://cran.r-project.org/package=broom.

6. Wickham, H., François, R., Henry, L. & Müller, K. dplyr: A Grammar of Data Manipulation. (2019).  <https://cran.r-project.org/package=dplyr>.

7. Fox, J. & Weisberg, S. *An R Companion to Applied Regression*. (Sage Publications, 2011).

**Supplementary Table S1**. Component loadings of questionnaire statements (items) in hyperactivity/impulsivity and inattention. One statement (Item 11: “It is likely to react hastily and that’s why it is failing tasks”) loaded equally on both components and was excluded from the analysis.

| **Item and Statement** | **Hyperactivity/impulsivity** | **Inattention** |
| --- | --- | --- |
| 1. My dog has a difficult time learning, because it is careless or other things can easily attract its attention. | 0.16 | **0.71** |
| 2. It's easy to attract its attention, but it loses its interest soon. | -0.36 | **0.96** |
| 3. It's difficult for it to concentrate on a task or play. | -0.02 | **0.85** |
| 4. It leaves from its place when it should stay. | 0.26 | **0.44** |
| 5. It cannot be quiet, it cannot be easily calmed. | **0.86** | -0.12 |
| 6. It fidgets all the time. | **0.83** | -0.06 |
| 7. It seems that it doesn't listen even if it knows that someone is speaking to it. | 0.14 | **0.56** |
| 8. It is excessive, difficult to control, and if it lunges it is hard to hold back. | **0.79** | 0.07 |
| 9. It would always play and run. | **0.75** | -0.17 |
| 10. It solves simple tasks easily, but it often has difficulties with complicated tasks, even if it knows them and has practiced them often. | 0.06 | **0.65** |
| 12. Its attention can be easily distracted. | 0.23 | **0.65** |
| 13. It cannot wait as it has no self-control. | **0.64** | 0.24 |

**Supplementary Table S2.** Variables derived from the behavioural questionnaire data.

| **Variable** | **Explanation** | **Possible values** |
| --- | --- | --- |
| Hyperactivity/impulsivity score | Continuous (response) variable. 5 statements concerning hyperactive/impulsive behaviour. Dog owners reported how often the statement is true for their dog on a 4-point Likert scale (from 1 = never to 4 = very often). Higher component scores indicate higher levels of hyperactivity/impulsivity. | -1.62 – 5.23 |
| Inattention score | Continuous (response) variable. 7 statements concerning inattentive behaviour. Dog owners reported how often the statement is true for their dog on a 4-point Likert scale (from 1 = never to 4 = very often). Higher component scores indicate higher levels of inattention. | -1.81 – 4.81 |
| Age | Continuous variable. The age of the dog (in years) at the time when the owner filled in the behavioural questionnaire. | 0.2 – 17.9 |
| Sex | Binomial variable. Sex of the dog. | 1: male  2: female |
| Breed | Categorical variable. Breed of the dog reported by the owner. 22 breeds with adequate sample sizes were chosen. Mixed breed was also included in the data. Individuals in other breeds were combined under ‘other’ breed group.  For Chihuahua, both coat types were combined.  For Chinese Crested Dog, both coat types were combined.  For Poodle, Toy, Miniature, and Medium sizes were combined. | Border Collie, Cairn Terrier, Chihuahua, Chinese Crested Dog, Coton de Tuléar, Finnish Lapponian Dog, German Shepherd, German Spitz Mittel, Golden Retriever, Jack Russell Terrier, Labrador Retriever, Lagotto Romagnolo, Lapponian Herder, Miniature Poodle, Miniature Schnauzer, Mixed breed, Other, Pembroke Welsh Corgi, Rough Collie, Shetland Sheepdog, Smooth Collie, Spanish Water Dog, Staff. Bull Terrier, Wheaten Terrier. |
| Sterilisation | Binomial variable. Information whether the dog was intact or neutered. | 0: intact  1: neutered |
| Activities/  training | Categorical variable. Describes how often the dog participated in activities or training. | 1: never/seldom  2: sometimes  3: at least weekly |
| Weaning age | Categorical variable. The age when the dog was weaned. Dogs still living with their mothers and dogs with missing information on weaning age were excluded. | 1: weaning < 7 weeks of age  2: weaning at 7 weeks of age  3: weaning at 8 weeks of age  4: weaning > 8 weeks of age |
| Body size | Categorical variable. Based on the average height of the breed, dogs were divided into three body size categories. | 1: small, ≤ 35 cm  2: medium, 36-49 cm  3: large ≥ 50 cm |
| Daily exercise | Categorical variable. The amount of dog’s daily exercise in hours. | 1: < 1 hour  2: 1-2 hours  3: 2-3 hours  4: > 3 hours. |
| Urban environment score | Continuous variable. Describes the environmental land-use around the current home of the dog. The coverages of three land-use types (artificial surfaces, agricultural areas, forests and semi-natural areas) were calculated within a 3-km range around the homes and simplified into a single continuous variable (higher values indicate a more urban environment). | -2.28 – 3.11 |
| Owner’s dog experience | Binomial variable. Describes the owner’s experience with dogs. | 1: the dog was the owner’s first dog  2: the dog was not the owner’s first dog |
| Daily time spent alone | Categorical variable. Describes the time that the dog spent alone daily at home without the presence of people. | 1: < 3 hours  2: 3-6 hours  3: 6-8 hours  4: > 8 hours. |
| Compulsive behaviour | Categorical variable. Describes how often the dog shows compulsive behaviours.  Subtraits: tail chasing, reflection/shadow snatching, surface licking, pacing, and staring (scale from 0 = never to 6 = several times per day); water bowl compulsion (scale from less than 5 minutes to 1 hour or more); self-biting (scale from 0 = never to 3 = several hours per day). | 0: low group  1: moderate group  2: high group |
| Fearfulness | Categorical variable. Describes how often the dog shows fear in different situations.  Subtraits: fear of strangers, fear of dogs, and fear of novel situations (scale from 0 = never to 5 = always). | 0: low group  1: moderate group  2: high group |
| Aggressiveness | Categorical variable. Describes the likelihood of the dog showing aggressive behaviour.  Subtraits: aggression towards strangers and towards family members (scale from 1 = never to 5 = always or almost always). | 0: low group  1: moderate group  2: high group |

**Supplementary Table S3.** The AIC model selection and the final models in generalised linear model analyses. N(hyperactivity/impulsivity) = 11 539; N(inattention) = 11 164.

| **Hyperactivity/impulsivity** | | | | | | | |  |  |
| --- | --- | --- | --- | --- | --- | --- | --- | --- | --- |
| Model | AIC | Fearfulness added | Compulsive behaviour added | Aggressiveness added | Breed added | Daily time spent alone added | Body size added | Daily exercise added | Owner’s dog experience added |
| Basic model (sex, age) | 15610 |  |  |  |  |  |  |  |  |
| Fearfulness | **15169** |  |  |  |  |  |  |  |  |
| Compulsive behaviour | 15197 | **14847** |  |  |  |  |  |  |  |
| Aggressiveness | 15393 | 15076 | **14783** |  |  |  |  |  |  |
| Breed | 15572 | 15112 | 14805 | **14735** |  |  |  |  |  |
| Daily time spent alone | 15596 | 15150 | 14827 | 14761 | **14714** |  |  |  |  |
| Body size | 15600 | 15147 | 14821 | 14751 | 14724 | **14704.22** |  |  |  |
| Daily exercise | 15565 | 15141 | 14833 | 14774 | 14720 | 14704.26 | **14693.85** |  |  |
| Owner’s dog experience | 15612 | 15168 | 14839 | 14773 | 14726 | 14704.26 | 14694.15 | **14683.04** |  |
| Sterilisation | 15591 | 15165 | 14845 | 14782 | 14734 | 14714 | 14704 | 14694 | 14682.17 |
| Weaning age | 15614 | 15172 | 14849 | 14786 | 14739 | 14719 | 14709 | 14698 | 14687 |
| Activities/training | 15603 | 15172 | 14850 | 14787 | 14738 | 14718 | 14708 | 14698 | 14687 |
| Urban environment score | 15612 | 15171 | 14847 | 14783 | 14736 | 14715 | 14705 | 14694 | 14684 |

Final model: sex, age, fearfulness, compulsive behaviour, aggressiveness, breed, daily time spent alone, body size, daily exercise, owner’s dog experience.

| **Inattention** |  |  |  |  |  |  |  |
| --- | --- | --- | --- | --- | --- | --- | --- |
| Model | AIC | Fearfulness added | Compulsive behaviour added | Breed added | Activities/  training added | Aggressiveness added | Daily time spent alone added |
| Basic model (sex, age) | 16917 |  |  |  |  |  |  |
| Fearfulness | **16682** |  |  |  |  |  |  |
| Compulsive behaviour | 16688 | **16497** |  |  |  |  |  |
| Breed | 16774 | 16531 | **16343** |  |  |  |  |
| Activities/training | 16774 | 16565 | 16396 | **16265** |  |  |  |
| Aggressiveness | 16801 | 16631 | 16464 | 16309 | **16233** |  |  |
| Daily time spent alone | 16913 | 16677 | 16493 | 16338 | 16259 | **16226.6** |  |
| Body size | 16910 | 16682 | 16497 | 16345 | 16264 | 16231 | 16225.03 |
| Sterilisation | 16903 | 16678 | 16495 | 16340 | 16263 | 16232 | 16226 |
| Weaning age | 16905 | 16676 | 16491 | 16341 | 16266 | 16234 | 16227 |
| Owner’s dog experience | 16917 | 16683 | 16499 | 16344 | 16266 | 16234 | 16227 |
| Daily exercise | 16885 | 16662 | 16487 | 16335 | 16262 | 16231 | 16227 |
| Urban environment score | 16917 | 16683 | 16499 | 16345 | 16267 | 16235 | 16228 |

Final model: sex, age, fearfulness, compulsive behaviour, breed, activities/training, aggressiveness, daily time spent alone.

**Supplementary Table S4.**Descriptive statistics. N(hyperactivity/impulsivity) = 11 539,

**N(inattention) = 11 164.**

| **Hyperactivity/impulsivity** | | | | |  | **Inattention** | | | |
| --- | --- | --- | --- | --- | --- | --- | --- | --- | --- |
|  |  | | **mean** | **SD** |  |  |  | **mean** | **SD** |
| Hyperactivity/ impulsivity score |  | | -0.01 | 1.00 |  | Inattention score |  | -0.01 | 0.98 |
| Age (years) |  | | 4.67 | 3.23 |  | Age (years) |  | 4.67 | 3.23 |
|  | | **N** | | **%** |  |  | | **N** | **%** |
| Sex | Female | 5896 | | 51.10 |  | Sex | Female | 5733 | 51.35 |
|  | Male | 5643 | | 48.90 |  |  | Male | 5431 | 48.65 |
| Breed | Border Collie | 240 | | 2.08 |  | Breed | Border Collie | 237 | 2.12 |
|  | Cairn Terrier | 94 | | 0.81 |  |  | Cairn Terrier | 84 | 0.75 |
|  | Chihuahua | 116 | | 1.01 |  |  | Chihuahua | 104 | 0.93 |
|  | Chinese Crested Dog | 137 | | 1.19 |  |  | Chinese Crested Dog | 130 | 1.16 |
|  | Coton de Tuléar | 111 | | 0.96 |  |  | Coton de Tuléar | 96 | 0.86 |
|  | Finnish Lapponian Dog | 461 | | 4.00 |  |  | Finnish Lapponian Dog | 420 | 3.76 |
|  | German Shepherd | 403 | | 3.49 |  |  | German Shepherd | 384 | 3.44 |
|  | German Spitz Mittel | 126 | | 1.09 |  |  | German Spitz Mittel | 119 | 1.07 |
|  | Golden Retriever | 154 | | 1.33 |  |  | Golden Retriever | 144 | 1.29 |
|  | Jack Russell Terrier | 153 | | 1.33 |  |  | Jack Russell Terrier | 129 | 1.16 |
|  | Labrador Retriever | 398 | | 3.45 |  |  | Labrador Retriever | 372 | 3.33 |
|  | Lagotto Romagnolo | 201 | | 1.74 |  |  | Lagotto Romagnolo | 190 | 1.70 |
|  | Lapponian Herder | 261 | | 2.26 |  |  | Lapponian Herder | 243 | 2.18 |
|  | Miniature poodle | 265 | | 2.30 |  |  | Miniature poodle | 241 | 2.16 |
|  | Miniature Schnauzer | 233 | | 2.02 |  |  | Miniature Schnauzer | 219 | 1.96 |
|  | Other | 6630 | | 57.46 |  |  | Mixed breed | 338 | 3.03 |
|  | Pembroke Welsh Corgi | 102 | | 0.88 |  |  | Other | 6249 | 55.97 |
|  | Rough Collie | 218 | | 1.89 |  |  | Pembroke Welsh Corgi | 92 | 0.82 |
|  | Shetland Sheepdog | 365 | | 3.16 |  |  | Rough Collie | 241 | 2.16 |
|  | Smooth Collie | 178 | | 1.54 |  |  | Shetland Sheepdog | 210 | 1.88 |
|  | Spanish Water Dog | 219 | | 1.90 |  |  | Smooth Collie | 345 | 3.09 |
|  | Staff. Bull Terrier | 181 | | 1.57 |  |  | Spanish Water Dog | 170 | 1.52 |
|  | Wheaten Terrier | 293 | | 2.54 |  |  | Staff. Bull Terrier | 210 | 1.88 |
| Body size | Small | 2952 | | 25.58 |  |  | Wheaten Terrier | 168 | 1.50 |
|  | Medium | 3920 | | 33.97 |  | Daily time spent alone | < 3 hours | 2827 | 25.32 |
|  | Large | 4667 | | 40.45 |  |  | 3-6 hours | 2979 | 26.68 |
| Owner’s dog experience | First dog | 3362 | | 29.14 |  |  | 6-8 hours | 3192 | 28.59 |
|  | Not a first dog | 8177 | | 70.86 |  |  | > 8 hours | 2166 | 19.40 |
|  |  |  | |  |  | Activities/training | Never/seldom | 2804 | 25.12 |
| Daily exercise | < 1 hour | 909 | | 7.88 |  |  | Sometimes | 3096 | 27.73 |
|  | 1-2 hours | 4772 | | 41.36 |  |  | At least weekly | 5264 | 47.15 |
|  | 3-4 hours | 4412 | | 38.24 |  | Fearfulness | Low | 4800 | 43.00 |
|  | > 3 hours | 1446 | | 12.53 |  |  | Moderate | 3172 | 28.41 |
| Daily time spent alone | < 3 hours | 2917 | | 25.28 |  |  | High | 3192 | 28.59 |
|  | 3-6 hours | 3047 | | 26.41 |  | Aggressiveness | Low | 6460 | 57.86 |
|  | 6-8 hours | 3330 | | 28.86 |  |  | Moderate | 3066 | 27.46 |
|  | > 8 hours | 2245 | | 19.46 |  |  | High | 1638 | 14.67 |
| Fearfulness | Low | 4986 | | 43.21 |  | Compulsive behaviour | Low | 3999 | 35.82 |
|  | Moderate | 3265 | | 28.30 |  |  | Moderate | 5372 | 48.12 |
|  | High | 3288 | | 28.49 |  |  | High | 1793 | 16.06 |
| Aggressiveness | Low | 6784 | | 58.79 |  |  |  |  |  |
|  | Moderate | 3138 | | 27.19 |  |  |  |  |  |
|  | High | 1617 | | 14.01 |  |  |  |  |  |
| Compulsive behaviour | Low | 4150 | | 35.96 |  |  |  |  |  |
|  | Moderate | 5530 | | 47.92 |  |  |  |  |  |
|  | High | 1859 | | 16.11 |  |  |  |  |  |
|  | | | | |  |  | | | |

**Supplementary Table S5.**Contrasts between different groups of categorical variables in the hyperactivity/impulsivity generalised linear model analysis. df = 1 in all comparisons.

| **Variable** | **Contrast** | **Z ratio** | **p** |
| --- | --- | --- | --- |
| **Sex** | Male vs. female | 8.27 | **<0.0001** |
| **Body size** | Large vs. small | 2.40 | **0.0166*** |
|  | Large vs. medium | -2.78 | **0.0224** |
|  | Medium vs. small | 4.74 | **0.0008** |
| **Daily time spent alone** | < 3 hours vs. 3-6 hours | 0.83 | 0.5471 |
|  | < 3 hours vs. 6-8 hours | -0.72 | 0.5979 |
|  | < 3 hours vs. > 8 hours | -3.99 | **0.0008** |
|  | 3-6 hours vs. 6-8 hours | -1.59 | 0.2159 |
|  | 3-6 hours vs. > 8 hours | -4.83 | **0.0008** |
|  | 6-8 hours vs. > 8 hours | -3.48 | **0.0031** |
| **Daily exercise** | < 1 hour vs. 1-2 hours | 2.50 | **0.0416** |
|  | < 1 hour vs. 2-3 hours | 3.96 | **0.0008** |
|  | < 1 hour vs. > 3 hours | 4.75 | **0.0008** |
|  | 1-2 hours vs. 2-3 hours | 2.66 | **0.0297** |
|  | 1-2 hours vs. > 3 hours | 3.74 | **0.0014** |
|  | 2-3 hours vs. > 3 hours | 1.94 | 0.1159 |
| **Owner’s dog experience** | The dog was owner’s first dog vs. the dog was not owner’s first dog | -4.65 | **0.0001** |
| **Compulsive behaviour** | Low vs. moderate | -11.61 | **0.0008** |
|  | Low vs. high | -21.50 | **<0.0001*** |
|  | Moderate vs. high | -13.78 | **0.0008** |
| **Aggressiveness** | Low vs. moderate | -6.80 | **0.0008** |
|  | Low vs. high | -10.21 | **<0.0001*** |
|  | Moderate vs. high | -4.71 | **0.0008** |
| **Fearfulness** | Low vs. moderate | -8.77 | **0.0008** |
|  | Low vs. high | -20.01 | **<0.0001*** |
|  | Moderate vs. high | -10.93 | **0.0008** |

P-values are controlled for false discovery rate if no *a priori* contrasts were set before analyses. Variables for which *a priori* contrasts were set and which p-values are not false discovery controlled are denoted with *. Significant effects are emboldened (p-value < 0.05). N = 11 539.

**Supplementary Table S6.**Contrasts between different groups of categorical variables in the inattention generalised linear model analysis. df = 1 in all comparisons.

| **Variable** | **Contrast** | **Z ratio** | **p** |
| --- | --- | --- | --- |
| **Sex** | Male vs. female | 8.95 | **< 0.0001** |
| **Daily time spent alone** | < 3 hours vs. 3-6 hours | 0.88 | 0.5114 |
|  | < 3 hours vs. 6-8 hours | -0.64 | 0.6190 |
|  | < 3 hours vs. > 8 hours | -2.99 | **0.0097** |
|  | 3-6 hours vs. 6-8 hours | -1.56 | 0.2003 |
|  | 3-6 hours vs. > 8 hours | -3.85 | **0.0005** |
|  | 6-8 hours vs. > 8 hours | -2.49 | **0.0327** |
| **Activities/training** | Never/seldom vs. sometimes | 0.88 | 0.5114 |
|  | Never/seldom vs.  at least weekly | 9.52 | **< 0.0001*** |
|  | Sometimes vs.  at least weekly | 9.28 | **0.0005** |
| **Compulsive behaviour** | Low vs. moderate | -12.17 | **0.0005** |
|  | Low vs. high | -17.04 | **0.0005** |
|  | Moderate vs. high | -8.63 | **0.0005** |
| **Aggressiveness** | Low vs. moderate | -5.93 | **0.0005** |
|  | Low vs. high | -7.50 | **0.0005** |
|  | Moderate vs. high | -2.72 | **0.0199** |
| **Fearfulness** | Low vs. moderate | -6.85 | **0.0005** |
|  | Low vs. high | -14.86 | **0.0005** |
|  | Moderate vs. high | -7.90 | **0.0005** |

P-values are controlled for false discovery rate if no *a priori* contrasts were set before analyses. Variables for which *a priori* contrasts were set and which p-values are not false discovery controlled are denoted with *. Significant effects are emboldened (p-value < 0.05). N=11 164.

Supplementary Figure legends:

Supplementary Figure S1.


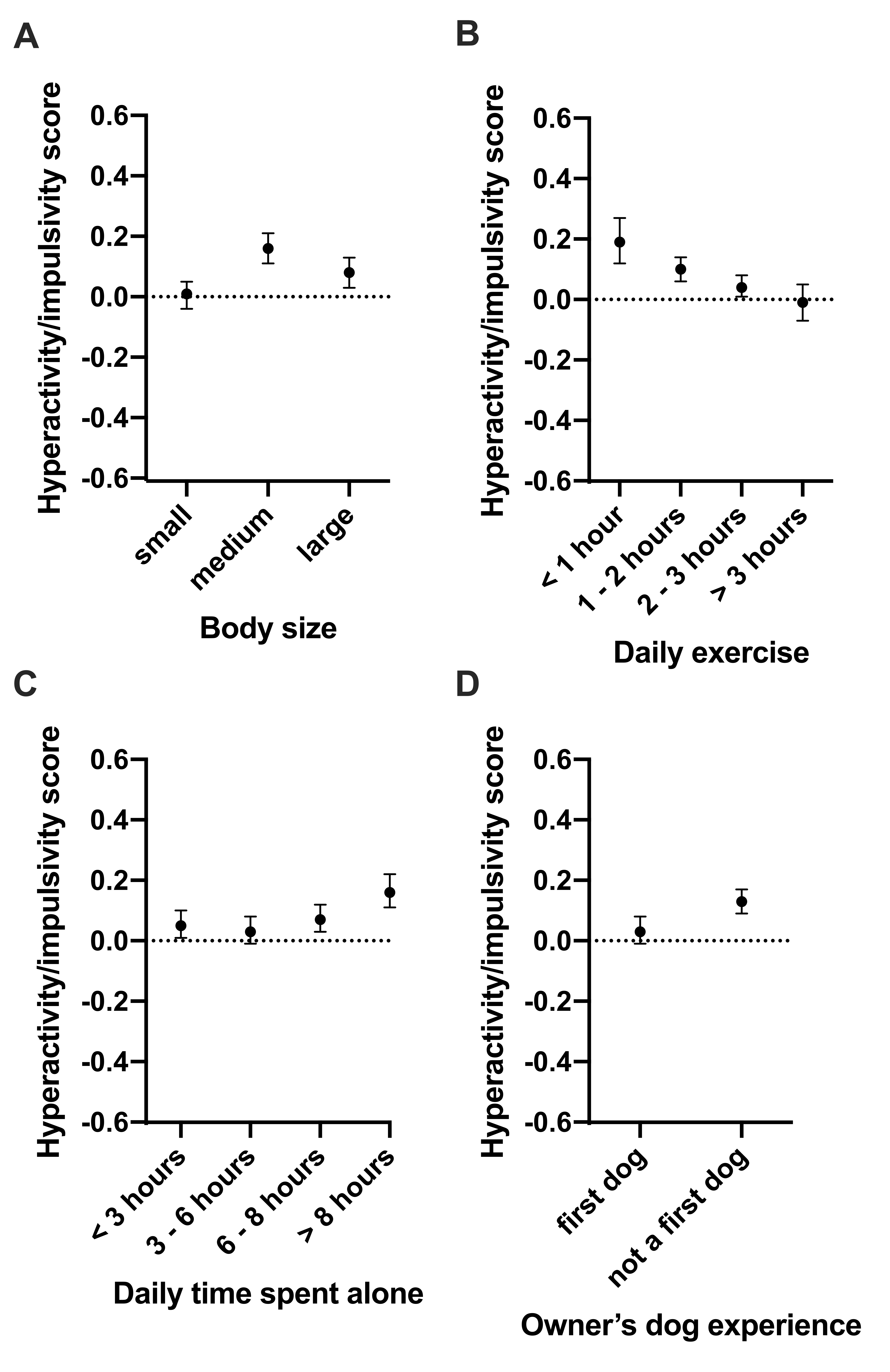


The effects of body size (A), daily exercise (B), daily time spent alone (C), and owner’s dog experience (D) on canine hyperactivity/impulsivity. Error bars indicate 95% confidence limits. N = 11 539.

Supplementary Figure S2.


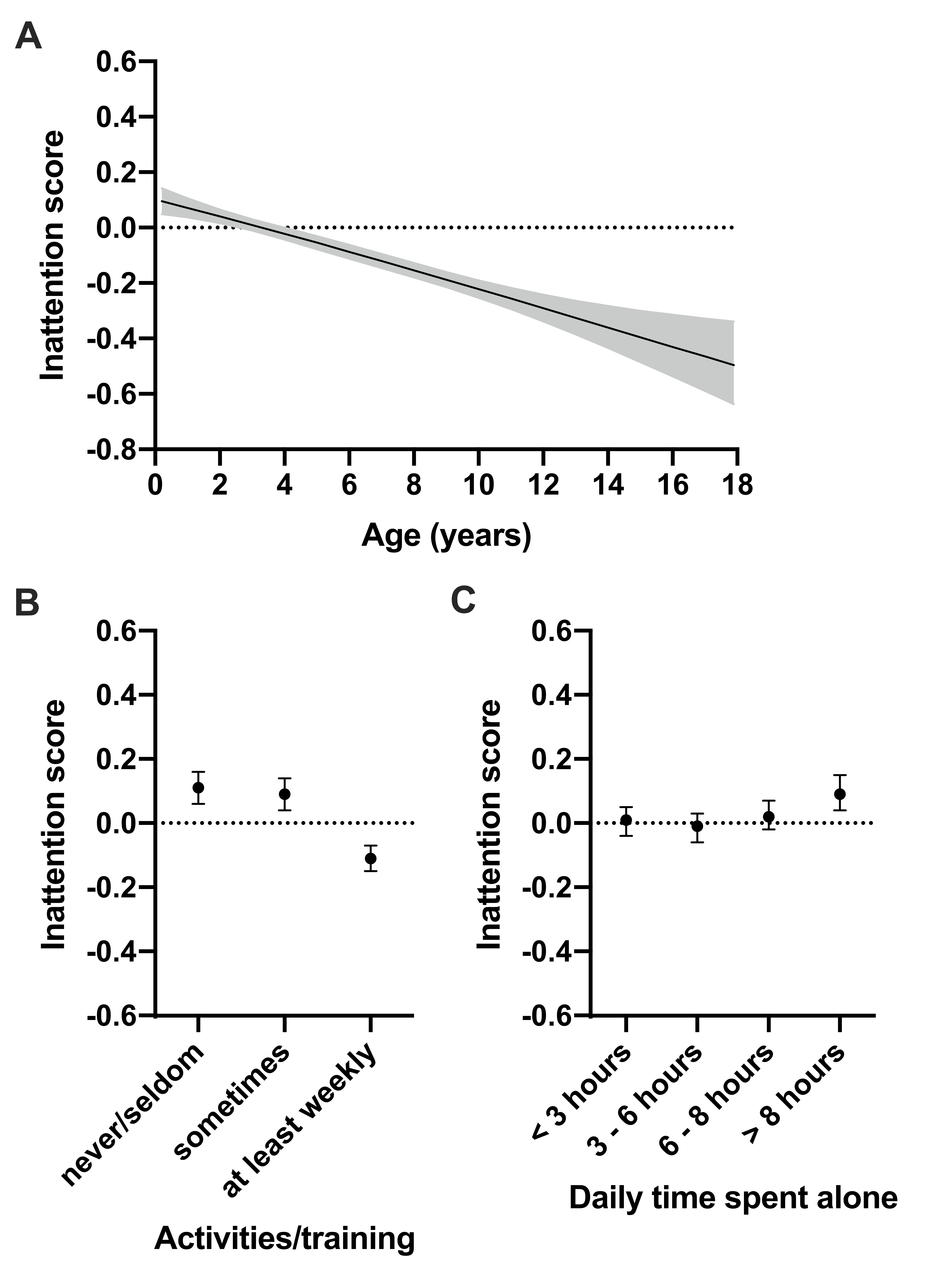


The effects of age (in years) (A), activities/training (B), and daily time spent alone (C) on canine inattention. Grey area (A) and error bars (B, C) indicate 95% confidence limits. N = 11 164.
